# Impact of pediatric tracheostomy on family caregivers’ burden and quality of life: a systematic review and meta-analysis

**DOI:** 10.3389/fpubh.2024.1485544

**Published:** 2025-01-15

**Authors:** Win Thu Aung, Natasha Yixuan Ong, Shina Qing Chun Yeo, Nur Syahindah Binti Juhari, Gwyneth Kong, Nicole-Ann Lim, Zubair Amin, Yvonne Peng Mei Ng

**Affiliations:** ^1^Ministry of Health Holding (MOHH), Singapore, Singapore; ^2^Yong Loo Lin School of Medicine, National University of Singapore, Singapore, Singapore; ^3^Department of Paediatrics, Yong Loo Lin School of Medicine, National University of Singapore, Singapore, Singapore; ^4^Department of Neonatology, Khoo Teck Puat-National University Children’s Medical Institute, National University Hospital, Singapore, Singapore

**Keywords:** psychological distress, financial burden, stress, coping, parents

## Abstract

**Introduction:**

The incidence of pediatric tracheostomy is on the rise. More children are undergoing tracheostomy at a younger age and living longer and cared for at home. Caring for children with tracheostomy affects the caregivers’ Quality of Life (QOL) and caregiver burden. We undertook a systematic review and meta-analysis to determine the impact of pediatric tracheostomy on caregivers’ QOL and caregiver burden.

**Methods:**

We performed a search for quantitative studies measuring QOL, caregiver burden and related factors such as psychological distress, coping, stress, and financial strain using validated instruments, reported by caregivers of children with tracheostomy. We searched PubMed, Embase, Cochrane Central Register of Clinical Trials, CINAHL, and PsycINFO with the following search terms: “pediatrics,” “tracheostomy,” “quality of life,” “caregivers,” “care burden” from the inception of respective databases to 23rd May 2024. Meta-analysis was conducted using R (version 4.3.1).

**Results:**

Twenty-three studies (1,299 caregivers) were included in systematic review. Seven studies (469 caregivers) using Pediatric Quality of Life Family Integrated Module underwent meta-analysis. The pooled mean total family impact score, parental health-related QOL, family functioning score were 70.29 [95% CI, 61.20–79.37], 69.27 [95% CI, 60.88–77.67], and 72.96 [95% CI, 65.92–80.00] respectively. Other key instruments were the Pediatric Tracheostomy Health Status Instrument and Zarit Burden Interview. Qualitative synthesis identified several risk factors for lower QOL and higher caregiver burden: comorbidities in children, younger age at tracheostomy, need for additional medical equipment, presence of older siblings, higher financial strain, being the sole caregiver or being unmarried, and maternal depression. Caregivers’ QOL correlated positively with coping and negatively with stress which is, in turn, associated with medical complications in the first year and the duration of tracheostomy. About 40% of mothers experienced moderate to severe caregiver burden while caring for their children with tracheostomy and this was significantly correlated with depression. Encouragingly, parents also reported positive experience including closeness of the family, feeling stronger, and having a strong sense of mastery.

**Discussion:**

Caregivers of children with tracheostomy experience low QOL and high caregiver burden, which were exacerbated by various medical and psychosocial factors. QOL should be assessed during clinical encounters to identify caregivers who require additional support which includes learning coping and stress reduction strategies.

**Systematic review registration:**

https://www.crd.york.ac.uk/prospero/display_record.php?RecordID=334457, identifier CRD42022334457.

## 1 Introduction

The incidence of pediatric tracheostomy is on the rise in many countries (1). The common indications for pediatric tracheostomy have shifted from treatment of acute airway obstruction to respiratory care of children with medically complex conditions. More children are undergoing tracheostomy at a younger age and living longer and cared for at home (2). After the hospital discharge, these children are looked after by family caregivers, typically their parents, with variable support from the healthcare services (3).

Caregivers of children with tracheostomies are required to perform multiple tasks at home, including cleaning, changing, suctioning the tracheostomy, and administering medications (4). These tasks are typically performed by trained professionals in hospital settings. Caregivers of a pediatric tracheostomy patient must remain in a constant state of vigilance, monitoring the child for emergencies such as accidental dislodgement, tube blockage by secretions, and equipment malfunctions. The duration of a tracheostomy varies depending on the child’s underlying medical condition. For certain conditions, such as neuromuscular disabilities, the need for a tracheostomy can be lifelong. In contrast, some pediatric conditions, such as chronic lung disease of prematurity, tend to improve over time, allowing for the successful reversal of the tracheostomy (5).

Caregiver burden is an individual’s measure of impact on their physical, psychological, emotional and financial wellbeing when taking care of the patient (6) and the caregiver’s perception of how well they are coping with their duties in response to the demands by the person receiving the care (7). Caregiver burden has many negative consequences on the wellbeing of the caregivers (8). For example, in a study involving caregivers of children with cerebral palsy, risk factors for caregiver burden were single parenthood, perceived ability to cope with caregiving, perceived family functioning, financial status, community support and child’s needs (9). In the context of tracheostomy care, caregiver burden affects several aspects of their wellbeing: physical (e.g., higher home care responsibilities), psychological (e.g., constant worry about emergencies), emotional (e.g., child’s inability to speak like other children) and financial (e.g., increased cost of frequent medical visits, consumables, and equipment) (10).

Quality of Life (QOL), as an overlapping concept to caregiver burden, is defined as an “individual’s perception of their position in life in the context of the culture and value systems in which they live in, and in relation to their goals, expectations, standards and concerns” (11). It is an important marker of functional abilities associated with an illness (12). A recent review determined that caregiver burden integrates the impact on all facets of caregiver wellbeing with higher specificity compared to QOL measures, while QOL is better at integrating different facets of caregiver wellbeing but with less specificity than caregiver burden (13).

A systematic review of adult patients with tracheostomies and their caregivers reported a range of mostly negative experiences related to the care, support, and management of a tracheostomy, speech and communication, wellbeing and QOL, disfigurement and body image, stigma and social withdrawal (14). Qualitative studies of caregivers of ventilator-dependent children, many with tracheostomies, reported high levels of caregiver stress, emotional strains, negative impact on family relationship, decision regret, living with daily threats of death, and need to devote extraordinary care and attention to their children’s need (15, 16).

However, there is a paucity of systematic reviews and meta-analyses on quantitative studies of QOL and burden experienced by caregivers of pediatric tracheostomy patients. Therefore, we undertook this systematic review and meta-analysis with the primary aim of synthesizing the effect of caring for tracheostomized children on caregivers’ QOL and caregiver burden. We chose to include both QOL and caregiver burden in our review to comprehensively capture various facets that can impact caregiver wellbeing. Our secondary aim is to identify factors associated with lower QOL and higher caregiver burden, in order to propose measures to improve caregiver QOL.

## 2 Methods

### 2.1 Search strategy

We conducted this systematic review and meta-analysis according to Preferred Reporting Items for Systematic Reviews and Meta-Analyses (PRISMA) (17) and Meta-analysis of Observational Studies in Epidemiology or MOOSE (Supplementary material 1) (18). We registered the protocol with the International Prospective Register of Systematic Reviews (PROSPERO) in June 2022 (PROSPERO registration: CRD42022334457).

We searched PubMed, Embase, Cochrane Central Register of Clinical Trials, CINAHL, and PsycINFO from the inception of respective databases to 23rd May 2024. We also searched grey literature and bibliography of included articles. The search strategy was devised in conjunction with a medical librarian with expertise in systematic review (Supplementary material 2).

### 2.2 Inclusion and exclusion criteria

We included studies reporting on quantitative QOL data or caregiver burden among caregivers of children with tracheostomy using validated instruments completed by caregivers. Studies investigating the relationship between caregiver burden and QOL with other related factors (e.g., psychological distress, coping, stress, financial burden) were included. We excluded articles that primarily studied cost of care, medical issues such as death and complications, outcomes of interventions, and validation of instruments. We also excluded review articles, conference reports, dissertations, abstracts, and articles in non-English languages.

### 2.3 Study selection and data extraction

Two review authors independently performed two-step process of screening—first by title and abstract, followed by full text of articles for study eligibility. Two review authors independently extracted relevant data from the included studies. We contacted authors of primary studies for missing data and clarifications. Discrepancies were resolved through team discussions.

### 2.4 Quality appraisal

Two review authors independently performed quality appraisal using a modified version of Newcastle-Ottawa Scale (NOS) for cross sectional studies (19).

### 2.5 Data synthesis and meta-analysis

We performed meta-analysis using R (version 4.3.1) if analyzable data were available from four or more studies. A random effects model was used due to heterogeneity of studies. We evaluated *I*^2^ statistics according to the Cochrane guidelines (0–40% = no heterogeneity; 30–60% = moderate heterogeneity; 50–90% = substantial heterogeneity; and 75–100% = considerable heterogeneity) (20). All studies underwent qualitative synthesis.

## 3 Results

**Figure 1:**
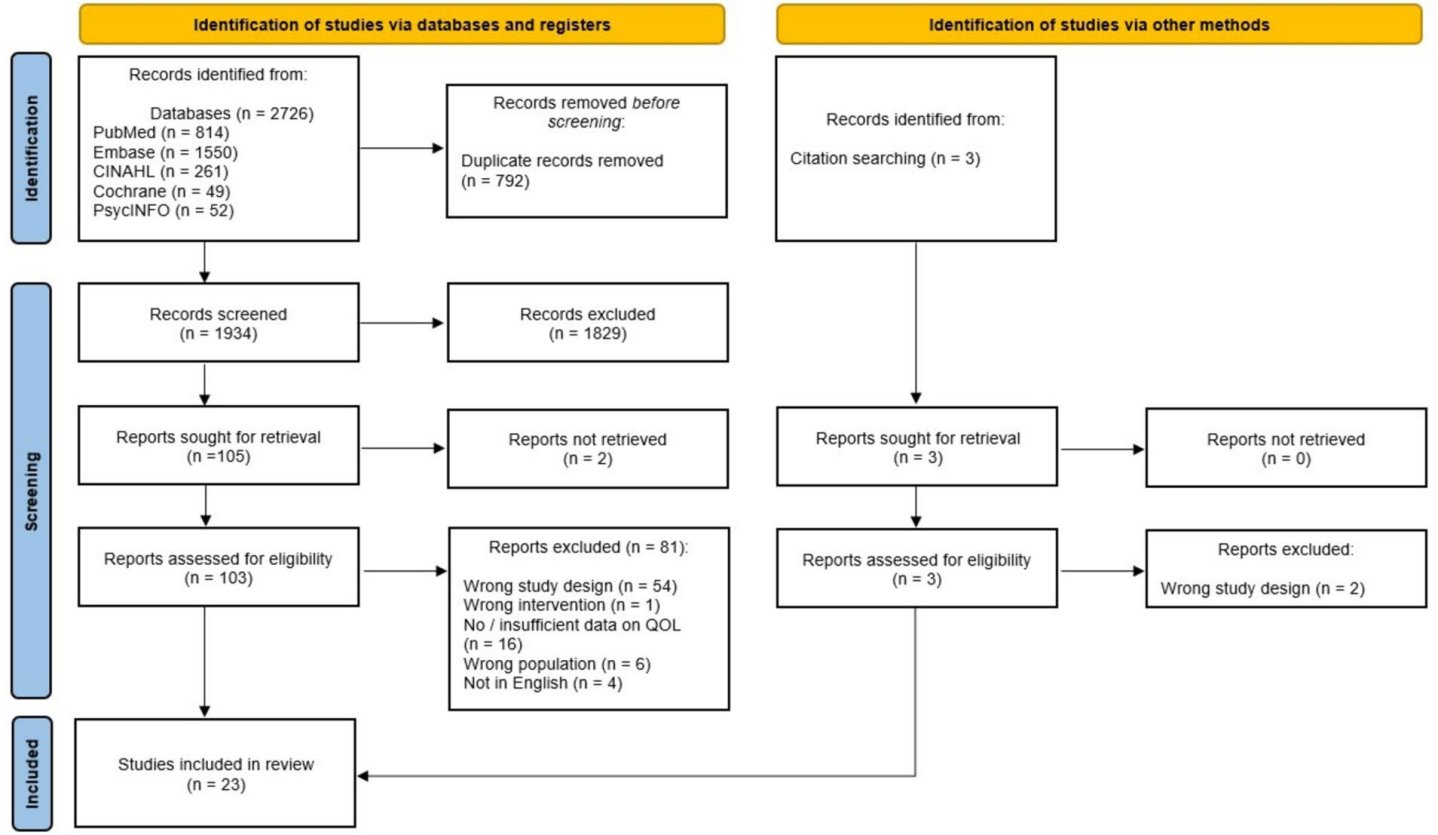
PRISMA flow diagram of the study selection process.

A total of 2,726 studies were found from database search. After de-duplication, title and abstract screening, 103 full text articles were retrieved. One article was found from citation search. Finally, 23 studies involving 1,299 caregivers were included in this review (Figure 1). Seven studies (469 caregivers) using Pediatric Quality of Life Family Integrated Module underwent meta-analysis.

We categorized all these studies as cross-sectional studies. Two studies, October et al. (21) and Wyning et al. (22), collected data at two time points but reported comparison using aggregated data (21) or a modeling method (22) instead of tracking individual participant’s data.

Table 1 displays the studies’ main characteristics which include country, study period, sample size, QOL tool, patient demographics, indication for and duration of tracheostomy and patient comorbidities. Table 2 describes the QOL instruments used, interpretation of scores, and studies which utilized the instruments.

**Table 1:** Summary of included studies.

First author, publication year, country, setting, study period	Sample size, instrument	Patient characteristics			
		Age (at survey or at tracheostomy), Mean (SD) or Median (Inter-quartile range) or Range	Indication (s) for tracheostomy	Duration of tracheostomy, Mean (SD) or Median (Inter-quartile range) or Range	Comorbidities
Chandran et al. (24), 2021, India, All India Institute of Medical Sciences, January 2015–June 2017	85, PedsQL 4.0 FIM	*Survey* Median 3.5 yrs. Range 9 mo – 14 years	Prolonged ventilation 69.4%, Upper airway obstruction 30.6%, Airway stenosis 20%, Respiratory papillomatosis 5.9%, Bilateral abductor cord palsy 4.7%	Median 2.5 years Range 8 mo to 4.5 years	Neurological impairment 49.4%, cardiorespiratory disease 16.4%, none 34.1%
Johnson et al. (25), 2021, United States, Children’s Medical Center Dallas, June 2018–June 2019	98, PedsQL 2.0 FIM	*Tracheostomy* Mean 1.6 (SD 3.5) years	Respiratory failure 78%; 55% were ventilator dependent	Not stated	Severe neurocognitive disabilities 36%
Salley et al. (26), 2021, United States, Children’s Medical Center Dallas, 2009–2019	13, PedsQL 4.0 FIM	*Tracheostomy* Mean 3.50 (SD 0.42) years	Airway obstruction 46.0%, Respiratory failure 75.7%, Pulmonary toilet 3.3%	Median 3.61 (SE 2.14) years	Not stated
Westwood et al. (23), 2019, United Kingdom, Sheffield Children’s Hospital, Not stated	25, PedsQL 4.0 FIM, PedsQL Generic Core Scale	*Survey* Median 6.25 years Range 0.5–16.5 years	Airway obstruction, neuromuscular conditions, complex congenital syndromes, severe facial burns	Median 3 years	Premature 24%, neurodevelopmental problem 28%, comorbid diagnoses with average 3 additional health problems 80% as a group
Liao et al. (27), 2021, United States, Children’s Medical Center Dallas, January 2015–December 2019	22, PedsQL 4.0 FIM	*Tracheostomy* Median 6.6 (IQR 42) months	Not stated	Not stated	Chronic respiratory failure 71%, short gestation 48%, newborn complications 48%, congenital malformations 41%
Mirza et al. (28), 2022, Saudi Arabia, 2010–2020	53, PedsQL 4.0 FIM, PedsQL Generic Core Scale	*Survey* Mean 6.85 (SD 4.19) years	Airway obstruction 58.5%, respiratory failure 34%, recurrent seizures 3.8% other 3.8%	Mean 3.64 (SD 3.37) years	Airway 56.6%, neurological 28.3%, cardiovascular 20.8%, genetic 13.2%, endocrine 11.3%, pulmonary 9.4%
Wynings et al. (22), 2023, United States, Children’s Medical Center Dallas, July 2019–July 2021	173 (66 caregivers at index admission, 107 caregivers at follow-up surveys) PedsQL 4.0 FIM	*Tracheostomy* Median 0.5 (IQR 1.1) years *Survey* Median 4.4 (IQR 6.6) years	Not stated	Not stated	Severe neurocognitive disability 68%
Din et al. (32), 2020, South Africa, Red Cross War Memorial Children’s Hospital, Not stated	68, PTHSI	*Tracheostomy* (83% had tracheostomy at <1 year of age)	Not stated	1 year 30.3%, 2–4 years 48.5%, 5 years 21.2%	Major comorbidities 57.4%
Hartnick et al. (12), 2003, United States, Children’s Hospital Medical Center in Cincinnati, Ohio, Not stated	154, PTHSI	Not stated	Not stated	<2 years 60%	Major comorbid medical problems 70% (neurological illness 40%, cardiopulmonary 35%, gastrointestinal 10%, neoplasm 15%)
Settoon et al. (33), 2021, United States, Louisiana State University Health Sciences Center, July 2019–October 2019	24, PTHSI	Not stated	Not stated	Not stated	Not stated
Hopkins et al. (30), 2009, United Kingdom, Central London Hospital, Not stated	26, PTHSI	*Survey* Mean 5 years Median 3 years	Subglottic stenosis 35%, bilateral vocal cord palsy 23%, severe tracheomalacia 19%, neurological deficits 12%	1–2 years 35%, 2–3 years 31%, 3 or more years 35%	Major conditions 50%
Al-Faleh et al. (34), 2023, Saudi Arabia Maternity and Children Hospital, September 2017–May 2022	24, PTHSI	Survey Mean 79 months	Not stated	Not stated	Major medical conditions (cardiac, neurological, pulmonary) 75%
Fuyuki et al. (35), 2021, Japan, Osaka Developmental Rehabilitation Center, May 2011–May 2018	21, J-ZBI_8	*Survey* Median 6 (IQR 0.50–26.8) years	Not stated	Not stated	Cerebral palsy 95%
Mavi et al. (36), 2021, Turkey, Istanbul Medeniyet University Faculty of Medicine Goztepe City Hospital, 2018–2020	26, ZBI, MBI, BDI, COPE	*Survey* Mean 6.69 (SD ±3.92) years	Chronic respiratory insufficiency 100%	Mean 25.46 (SD 31.45) months	Cerebral palsy 100%
Yotani et al. (37), 2014, Japan, 2 children’s hospitals and 6 rehabilitation centers in Tama Health Care Network, August 2011–March 2013	14, ZBI	*Survey* Mean 15.4 (SD ±11.8) years Classified into 2 age groups: Younger group mean 5.9 (SD ±3.6) years; Older group mean 27.4 (SD ±6.1) years	Not stated	Not stated	In younger group, Cerebral palsy 24%, congenital anomaly syndrome 21%, hypoxic encephalopathy 11%, refractory epilepsy 16%, sequelae of acute encephalopathy 11%, brain malformation 5%, other 13%
Gursoy et al. (38), 2022, Turkey, 4 Pediatric pulmonology centers, 2017–2019	85, ZBI, MBI, BDI, RSES	*Survey* Median 4 (IQR 2–13) years	Not stated	Mean 2.0 (SD 0.2) years	Neurological diseases 37.6%, chronic lung diseases 29.6%, metabolic diseases 17.7%, heart diseases 7.0%, chromosomal anomalies 3.5%, craniofacial anomalies 2.3%, airway anomalies 2.3%
Joseph et al. (39), 2014, United States, Duquesne University and Nemours/Alfred I. duPont Hospital for Children, Not stated	71, FILE, F-COPES, PGWBI	*Survey* Mean 21.6 (SD ±10.0) months *Tracheostomy* Mean 3.24 (SD ±2.3) months	Not stated	Mean 18.22 (SD 9.59) months	Not stated
Montagnino et al. (40), 2004, United States, Texas Children’s Hospital, January 2000–December 2001	50, IOFS, F-COPES	*Tracheostomy* Range 2 weeks - 14 years 72% 2 weeks-1 year old 28% >1 year old	Bronchopulmonary dysplasia 38.8%, encephalopathy 22%, myelomeningocele 11%, Pierre-Robin syndrome 11%, arthrogryposis 5.5%, diaphragmatic hernia 5.5%, Escobar syndrome 5.5%, central hypoventilation syndrome 11.1%	Not stated	Gastroesophageal reflux disease 72%
Singer et al. (42), 1989, United States, Rainbow Babies & Children’s Hospital, Not stated	27, Modified IOFS	*Survey* Mean 4.0 (SD ±2.6) years	Very low birthweight 27%, medical complications at birth 79%, neurological complications 48%	Mean 32.7 (SD 32) months	Not stated
October et al. (21), 2020; United States, Children’s National Health System, January 2015 to December 2017	25, ACQLQ	*Survey* Median 11 (IQR 0–248) month	Respiratory 56.4%, Non-respiratory 43.6%	Not stated	Not stated
Baddour et al. (41), 2021, United States, Children’s Hospital of Pittsburgh, 2009–2018	45, COST, FDQ, BSFC-s	*Survey* Median 6 Range 1.8–17 years *Tracheostomy* Median 5 Range 0–180 months	Airway condition 53.3%, lung condition 55.6%, neuromuscular condition 31.1%, brain condition 40.0%, spinal cord condition 11.1%, other 17.8%	Not stated	Not stated
Verstraete et al. (31), 2023, South Africa	144, PedsQL, General Rating of Health, EQ-5D-5L, PTHSI, CarerQoL	*Survey* Mean 5.9 years	Upper airway obstruction 59.0%, neuromuscular disease 17%, long-term ventilation 10.0%	Not stated	Not stated
Koker et al. (43), 2023, Turkey January 2011–December 2021	26, FSS, WHOQoL-BREF	*Survey* Mean 102 months	Prolonged ventilation 65.4%	Mean 22 days	Neuromuscular 30.8%, neurometabolic disease 26.9%

IQR, Interquartile Range; SD, Standard Deviation; SE, Standard Error; PedsQL, Pediatric Quality of Life; FIM, Family Impact Module; PTHSI, Pediatric Tracheostomy Health Status Instrument; J-ZBI_8, Japanese version of the Zarit Caregiver Burden Interview; BDI, Beck Depression Inventory; MBI, Maslach Burnout Inventory; COPE, Coping Orientation to Problems Experienced; RSES, Rosenberg Self-esteem Scale; FILE, Family Inventory of Life Events and Changes; F-COPES, Family Crisis Oriented Personal Evaluation Scale; PGWBI, Psychological General Well Being Index; IOFS, Impact on Family Scale; ACQLQ, Adult Carer Quality of Life Questionnaire; COST, Comprehensive Score for Financial Toxicity; FDQ, Financial Distress Questionnaire; BSFC-s, Burden Scale for Family Caregivers-short version; FSS, Functional Status Scale; WHOQOL-BREF, World Health Organization Quality-of-Life Scale.

**Table 2:** Description of instruments used in the studies and interpretation of the scores.

Instrument, reference	Measurements and descriptions	Scoring	Included studies
Pediatric Quality of Life (PedsQL) Family Impact Module (FIM) Varni et al. (29), 2004	36-items, 8 domains. Physical functioning (6 items) Emotional functioning (5 items) Social functioning (4 items) Cognitive functioning (5 items) Communication (3 items) Worry (5 items) Daily activities (3 items) Family relationships (5 items)	5-point Likert scale from 0 (never) to 4 (almost always), reverse scored and transformed to a 0–100 scale, higher scores denoted better functioning. Three scores are derived: Total Family Impact Module score: sum of all 36 items Caregiver HRQOL Summary Score: 20 items in the Physical, Emotional, Social, and Cognitive Functioning Scales Family Functioning Summary Score: 8 items in the Daily Activities and Family Relationships Scales	Chandran et al. (24), 2021 Johnson et al. (25), 2021 Salley et al. (26), 2021 Westwood et al. (23), 2019 Liao et al. (27), 2021 Mirza et al. (28), 2022 Wynings et al. (22), 2023
Adult Carer Quality of Life Questionnaire (AC-QOL)	40-items; 8 domains Support for caring (5 items) Caring choice (5 items) Caring stress (5 items) Money matters (5 items) Personal growth (5 items) Sense of value (5 items) Ability to care (5 items) Carer satisfaction (5 items)	4 points scoring (never; some of the time; a lot of times, always); some items are scored in reverse. Scored from 0 to 120. 0–40: Low quality of life 41–80: Mid-range quality of life 81 and above: High quality of life	October et al. (21), 2020
Pediatric Tracheostomy Health Status Instrument (PTHSI)	34-items, 4 domains. Specifically developed for tracheostomy patients and their caregivers. Physical symptoms of the child (7 items) Medical visits and costs (3 items) Stress and coping (caregiver’s viewpoint of child’s perspective) (3 items) 4. Stress and coping (caregiver’s own perspective) (17 items)	5-point Likert scale from 1 to 5. The total score can range from 0 to 150, with higher scores indicating a better QOL.	Din et al. (32), 2020 Hartnick et al. (44), 2003 Settoon et al. (33), 2021 Hopkins et al. (30), 2009 Al-Faleh et al. (34), 2023 Verstraete et al. (31), 2023
Zarit Caregiver Burden Scale (ZCBS) or Zarit Burden Interview (ZBI)	22-items, 5 domains. Assesses stress experienced by caregivers with patients in need of care. Mental tension/ Deterioration of private life Irritability/ Restriction Deterioration in Social relations Economic burden Dependence	5-point Likert scale from 0 (never) to 4 (nearly always). The total scores can range from 0 to 88, with higher scores indicating higher burden. The scores are categorized as follows: 0–20 points: no burden 21–40 points: mild burden 41–60 points: moderate burden 64–88 points: severe burden	Fuyuki et al. (35), 2021 Mavi et al. (36), 2021 Yotani et al. (37), 2014 Gursoy et al. (38), 2022
Family Crisis Oriented Personal Evaluation Scales, F-COPES	30-items, 5 subscales. Assesses coping strategies used by primary caregiver. Acquiring social support Reframing Seeking spiritual support Mobilizing family to acquire and accept help Passive appraisal	5-point Likert scale from 1 (strongly disagree) to 5 (strongly agree). Total score ranges from 30 to 150. Higher scores indicate higher level of coping.	Joseph et al. (39), 2014 Montagnino et al. (40), 2004
Impact on Family Scale, IOFS	33-items, 4 subscales. Measures the impact of caring for an unwell child as perceived by the main caregiver. Financial support Disruption of social relations General negative impact Coping	4-point Likert scale from 1 (strongly agree) to 4 (strongly disagree). Four subscale scores and a total score were obtained. The total score, as well as three subscales (Financial support, Disruption of Social Relations, General Negative impact) measure the negative impact, hence a higher score indicates more negative impact on the family. For the Coping subscale, a higher score indicates a positive impact.	Montagnino et al. (40), 2004 Singer et al. (42), 1989
Family Inventory of Life Events and Changes, FILE	71-items. Assesses normative and nonnormative life events that parents go through in the past 12 months	Dichotomous scale: yes or no The score ranges from 0 to 71, with higher scores indicate higher stress.	Joseph et al. (39), 2014
Psychological General Well Being Index, PGWBI	22-items. Assesses intrapersonal affective or emotional states	5-point Likert scale from 0 to 5, and the global score ranges from 0 to 110. 0–60: severe distress 61–72: moderate distress 73–110: positive wellbeing.	Joseph et al. (39), 2014
Comprehensive Score for Financial Toxicity, COST	11-items. Assesses subjective experience of Financial Toxicity	5-point Likert scale from 0 (not at all) to 4 (very much). The score ranges from 0 to 44, with lower scores indicating worse financial toxicity.	Baddour et al. (41), 2021
Financial Distress Questionnaire, FDQ	2-items. Evaluates the severity of financial toxicity	There are three ordinal grade categories: Grade 1: mild Grade 2: moderate Grade 3: severe Thereafter, the grades are assessed as a dichotomized as either low (Grade 1) or high (Grade 2 and 3) financial toxicity.	Baddour et al. (41), 2021
Burden Scale for Family Caregivers-short version, BSFC-S	10-items. Assess the burden of care	4-point Likert scale from 0 (strongly disagree) to 3 (strongly agree). The score ranges from 0 to 30, with higher scores denoting greater caregiver burden. It can be categorized as: 0–4: no to mild burden of care 5–14: moderate burden 15–30: severe to very severe	Baddour et al. (41), 2021
EQ-TIPS	Assesses six dimensions—movement, play, pain, communication, social interaction and eating	Each dimension has 3 response levels—no problems, some problems, a lot of problems	Verstraete (31), 2023
EQ-5D-5L South African version	Assesses five dimensions—mobility, self-care, usual activities, pain/discomfort, anxiety/depression	Each dimension has 5 response levels—no problems, slight problems, moderate problems, severe problems, unable to/ extreme problems. Each response level is converted to a number, and the total score is written as a five-digit code with each digit corresponding to the response level for respective domain.	Verstraete (31) 2023
World Health Organization Quality-of-Life Scale (WHOQOL-BREF)	27 items. Assesses QOL of caregivers with 5 domains—physical, occupational, social, psychological, and economic	5-point Likert scale from 1 to 5	Koker et al. (43), 2023
Functional Status Scale (FSS)	Assesses functional (emotional, mental, and motor) and ongoing nutritional, respiratory, and communicative status of patients post-discharge	5-point Likert scale from 1 to 5 Higher scores indicate higher functional disorder.	Koker et al. (43), 2023

### 3.1 Quality of the studies

The quality of 22 studies were good or very good, and one was satisfactory (Supplementary material 3). We included all studies in the review.

#### 3.1.1 Meta-analysis of studies using pediatric quality of life family impact module (PedsQL FIM)

We performed meta-analysis on PedsQL FIM scores from seven studies involving 469 caregivers (22–28).

**Figure 2:**
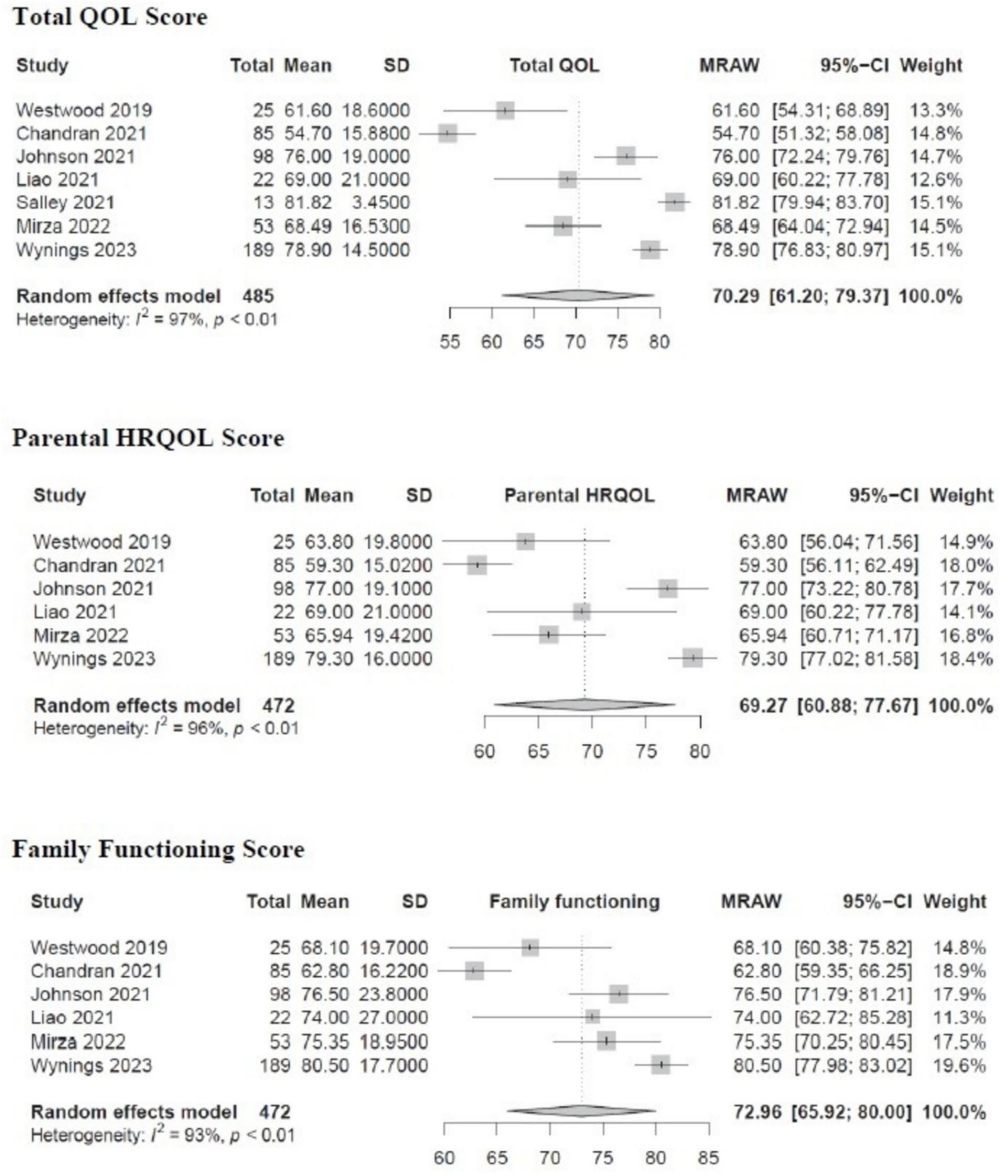
Pooled analysis of total QOL, parental HRQOL and family functioning scores in PedsQL FIM studies.

Figure 2 shows the results of meta-analysis on three summary scores. The pooled mean total family impact score was 70.29 (95% CI, 61.20–79.37), mean parental health-related QOL was 69.27 (95% CI, 60.88–77.67), and mean family functioning score (average of 8 items under daily activities and family relationships domains) was 72.96 (95% CI, 65.92–80.00). There was substantial heterogeneity between the studies (*I*^2^ = 93–97%, *p* < 0.01). As a comparison, the PedsQL FIM validation study on medically fragile children with complex chronic diseases in the home setting reported mean total family impact score of 62.49 (SD 17.26); parental health related QOL of 62.94 (SD 19.83); and family functioning score of 68.81 (SD 24.11) (29).

Supplementary material 4 displays the results of meta-analysis on the eight PedsQL FIM subdomains. The subdomain with the lowest score was worry 61.90 (95% CI, 51.69–72.11), followed by daily activities 64.60 (95% CI, 55.12–74.07). Corresponding scores from PedsQL FIM validation study were: worry 56.82 (SD 25.52) and daily activities 51.89 (SD 31.48) (29). The subdomains with the highest scores were cognitive functioning 80.92 (95% CI, 76.91–84.93) and family relationship 80.38 (95% CI, 70.36–90.40) with the corresponding scores from the validation study: cognitive functioning 74.09 (SD 18.95) and family relationship 78.95 (SD 27.62) (29). There was substantial heterogeneity between the studies (*I*^2^ = 79–99%, *p* < 0.01).

The result from our meta-analysis show higher values in total and sub-domain scores than corresponding values from the original PedsQL FIM validation study by Varni et al. (29). There are several plausible reasons for this finding. The validation study was conducted among families with medically fragile children with complex medical conditions; whereas, more families in this meta-analysis had children with fewer medical comorbidities. Secondly, the validation study was performed in early 2000s when medical care and support system might not have been well developed, whereas studies in this meta-analysis were more recent, published in the last 5 years. Nevertheless, the scores from our meta-analysis are similar to family caregivers caring for children with other chronic medical conditions such as sickle cell diseases, nephrotic syndrome, childhood cancers and congenital cyanotic heart disease (Supplementary material 5) indicating both groups of family caregivers experience low QOL.

#### 3.1.2 Qualitative synthesis of PedsQL FIM studies

Chandran et al. determined that caregivers performed well in the cognitive functioning subdomain. Despite lower scores in other areas, caregiving seemed to have less impact on caregivers’ concentration, memory, and thought processes (24). Salley et al. reported higher overall QOL scores, with the family relationship subdomain scoring the highest (26). However, this finding might be influenced by response bias due to a small sample size (only 13 out of 337 parents participated) (26). Similarly, Westwood et al. also reported higher scores in cognitive functioning and family relationships (23).

Chandran et al. (24) and Johnson et al. (25) found no significant association between indications for tracheostomy and QOL scores. Chandran et al. highlighted significantly poorer QOL across all three summary scores (total family impact score, parental Health-Related QOL (HR-QOL) and family functioning score) in caregivers of children with co-morbidities. Additionally, caregivers of children ≤6 years old had lower parental HR-QOL and family functioning scores (24). Liao et al. observed no significant differences in scores between socioeconomically ‘less disadvantaged’ and ‘more disadvantaged’ groups (27). Wynings et al. studied the largest cohort (255 patients) and found that emotional functioning improved over time (22). Caregivers’ wellbeing had the most significant impact on QOL, regardless of the child’s underlying conditions (22). Mirza et al. found that the highest scores were in cognitive functioning, followed by communication. Within the family functioning summary score, which consists of two domains, parents reported higher scores in family relationships than in daily activities. This indicates that while parents are burdened with the additional daily activities required to care for children with tracheostomies, their family relationships remain better preserved (28).

In summary, these studies show that caregivers of tracheostomized children have better QOL in the cognitive functioning and family relationships subdomains. There is no association between tracheostomy indications and QOL scores. However, caregivers of younger children or those with co-morbidities tend to have lower QOL and family functioning scores.

### 3.2 Qualitative synthesis of remaining studies measuring QOL and caregiver burden

We performed qualitative synthesis on the remaining 16 studies involving 830 caregivers. Six studies (12, 30–34) used the Pediatric Tracheostomy Health Status Instrument (PTHSI), four studies (35–38) used the Zarit Burden Interview (ZBI) which is also known as Zarit Caregiver Burden Score (ZCBS), and six studies (21, 39–43) used a combination of other instruments. We could not perform any meta-analysis due to incomplete data and heterogeneity of the instruments used.

#### 3.2.1 Studies based on PTHSI

PTHSI is a specific tool to assess QOL of tracheotomized children in four domains: physical symptoms of the child, medical visits and cost, caregiver’s viewpoint of child’s psychological health, and parent’s perspective of caregiver burden (44). The last domain represents caregiver burden which is analyzed in this review.

Hartnick et al. (12) and Hopkins et al. (30) found that caregiver burden was significantly related to the parent’s perception of the child’s health and the economic cost of care (12). Din et al. (32), Settoon et al. (33), and Al-Faleh et al. (34) found that parents of tracheotomized children with major medical conditions experienced a higher burden, but this burden did not differ according to family income. Verstraete et al. found that many caregivers derived some fulfillment from caring for their children, but were unable to focus on their own needs (31). Overall, these studies suggest that caregivers’ QOL is deeply intertwined with their children’s illness severity and QOL, indicating an improvement in children’s health status may have a positive impact on their parents’ QOL.

#### 3.2.2 Studies based on ZBI

Fuyuki et al. found that the quality of patient’s relationship with other family members besides the main caregiver was better in low care burden groups than in high care burden groups, suggesting the level of care burden can affect familial relationships (35). Yotani et al. reported age-related differences in caregiver burden. For caregivers of younger patients (<15 years), there was no link between caregiver burden and need for home mechanical ventilation. However, caregiver burden increased with the presence of older siblings, indicating that family dynamics play a role in caregiver stress (37).

Mavi et al. studied mothers of children with cerebral palsy and chronic respiratory insufficiency and identified differences in coping mechanisms. Mothers of children without tracheostomy showed more active coping, utilized emotional/social support networks, and demonstrated more acceptance than mothers of children with tracheostomy (36). Gursoy et al. examined the correlation between caregiver burden and mental health (38). They found that 40% of mothers experienced moderate to severe caregiver burden, and this was significantly correlated with depression. Caregiver burden did not significantly differ based on maternal education, occupation, tracheostomy duration, or child’s age (38).

#### 3.2.3 Studies using other instruments

Joseph et al. studied the impact of stress and coping on caregiver’s QOL (39). They found that caregiver QOL correlated positively with coping strategies and negatively with stress levels, indicating that effective coping mechanisms can mitigate the distress experienced by caregivers. Singer et al. assessed the impact of a tracheotomized child’s disability on family life and maternal perception of stress (42). There was a significant association between financial stress, younger age at tracheostomy, and medical complications during the first year. Maternal stress was associated with medical complications in the first year and the duration of tracheostomy. Encouragingly, parents also reported positive experience including closeness of the family, feeling stronger, and having a strong sense of mastery (42)—similar to findings in meta-analysis of PedsQL FIM studies which demonstrated high family functioning score.

Montagnino et al. found a positive correlation between a family’s economic status and their ability to access community resources, suggesting that financial stability can enhance support for caregivers (40). October et al. reported minimal changes in caregiver QOL scores shortly after the decision for tracheostomy, implying that caregivers may need time to adjust to the new care demands (21). Baddour et al. explored the concept of financial toxicity and determined higher financial toxicity was associated with increased caregiver burden (41). Lastly, Koker et al. found that a child’s worsening functional status negatively affected caregiver QOL across various domains (43).

In summary, these studies highlight how the complex interplay of stress, coping mechanisms, financial stability, adjustment period, and the child’s functional status influence the QOL of caregivers of children with tracheostomy. They underscore the need for comprehensive support systems and targeted interventions to improve caregivers’ QOL.

## 4 Discussion

To the best of our knowledge, this is the first comprehensive systematic review and meta-analysis on caregiving burden and QOL of family caregivers of children with tracheostomy. Caring for young children with tracheostomy negatively affects caregivers’ QOL, imposes additional burden on them, results in high level of stress and depression, and impairs their family functioning. Among the subdomains, ‘worry’ and ‘daily activities’ were the most negatively affected domains, while ‘cognitive functioning’ and ‘family relationship’ were the least affected domains.

Risk factors for poorer QOL and higher caregiver burden include caring for children with associated comorbidities (12, 24, 32), younger age at tracheostomy (24), poor functional status (43), need for additional therapeutic interventions (35), having a healthy school-going older sibling (37), higher financial strain (41), being the sole caregiver and being unmarried (41), and maternal depression (38). Indication for tracheostomy (24, 25, 28) and duration of tracheostomy (32, 38) did not have a consistent association with caregiver’s QOL and caregiver burden. Moderate and severe caregiver burden was associated with maternal depression, but not correlated with educational level, duration of tracheostomy and age of children (38).

We found correlations between stress, coping, and caregiver burden. Previous studies in caregivers of medically vulnerable children established a close link between caregivers’ wellbeing and health, and psychosocial outcomes which included higher risks of child abuse and neglect (45). Tracheostomy adversely impacts family’s finances, employment opportunity of the family caregivers, and the family’s ability to provide required services to the patients (41), which can result in suboptimal care of the tracheotomized child. Conversely, higher QOL scores are associated with better coping and lower stress levels (39) and improved psychosocial health in caregivers (28).

Although worry was the worst affected sub-domain, it is encouraging to note that the study with the largest cohort reported improvement in worry and emotional functioning over time (28). Family caregivers of a child with tracheostomy struggle to balance performing multiple tasks associated with tracheostomy care while assuming the typical role of parents. However, parents’ worry lessens as they become more competent with tracheostomy care and when provided with additional support. As psychosocial health has the largest impact on caregivers’ QOL (28), medical providers should target interventions to allay parental anxiety and worry and counsel them about active coping strategies.

Measurements of both generic QOL and disease/condition specific-QOL are useful. Generic QOL instruments allow comparison with caregivers of healthy children or children with other chronic medical conditions. Condition specific QOL instruments can provide deeper and relevant insights into caregiver experiences (44). For example, studies using PTHSI revealed associations between caregiver burden with cost of care (12), parental perception of child’s health (12), and caregiver’s perspective of child’s QOL (30) which may not be captured efficiently by generic instruments.

### 4.1 Strengths and limitations

We included both caregiver burden and QOL to broadly assess the impact of tracheostomy on family caregivers. Our work builds up on the knowledge base synthesized in a recent systematic review by Acorda et al. whose primary objective was to identify instruments used to measure psychosocial outcomes of caregivers of children with tracheostomy (46). In comparison, our principal objective was to synthesize the findings from primary studies with a secondary objectives of identifying factors affecting caregiver burden and QOL. Our review includes additional primary studies and a meta-analysis.

We would like to highlight several limitations. Studies were heterogeneous due to the variations in instruments used, age of patients, indications and duration of tracheostomy, and presence of concomitant conditions. Majority of caregivers were females (i.e., mothers) with underrepresentation of other family caregivers. There was a paucity of studies that evaluated QOL longitudinally over time. Our review focused on parental QOL and caregiver burden and did not explore the effect of tracheostomy on issues such as disfigurement, body-image, self-confidence, guilt and social isolation which have been reported among adults with tracheostomy (14).

### 4.2 Implications for practice, research and education

Our review suggests that assessment of QOL and caregiver burden should be a routine part of comprehensive assessment of tracheostomy patients at every touch point. Findings from this review can be used to counsel parents in preparing for their child’s tracheostomy and aid in the process of consent taking.

Based on our review, we identified the following risk factors of poorer caregiver QOL: presence of associated comorbidities in the children, early age of tracheostomy, longer duration of tracheostomy and a lack of social support. The risk factors for higher caregiver burden include need for additional medical equipment, older patients with home mechanical ventilation, having a healthy school going older sibling, being the sole caregiver and being unmarried. These risk factors can be used as a screening tool by healthcare providers to identify caregivers at high risk for poor QOL and caregiver burden (Table 3). As caregivers are integral to the patient’s recovery; efforts should be made to ensure caregiver’s coping so that they feel supported in this journey.

**Table 3:** Screening tool for providers to identify caregivers at risk of poor QOL and caregiver burden.

**Poorer Quality of Life** Presence of associated comorbidities in children Early age of tracheostomy Longer duration of tracheostomy Lack of social support **Higher Caregiver Burden** Need for additional medical equipment Older tracheotomized patients with home mechanical ventilation Having a healthy school going older sibling Being the sole caregiver and being unmarried

### 4.3 Conclusion

We suggest that longitudinal assessment of QOL and caregiver burden should be routinely performed for comprehensive management of children with tracheostomy. This will identify caregivers who need additional assistance and enable relevant stakeholders to implement targeted interventions to improve caregiver’s QOL. Both generic QOL tools and tracheostomy-specific QOL tools are useful to assess parental QOL. We also recommend teaching family caregivers coping and stress reduction strategies, as better coping and reduced stress result in higher caregiver QOL, which in turn may improve family’s wellbeing and outcomes of children with tracheostomies.

## Data Availability

The original contributions presented in the study are included in the article/Supplementary material, further inquiries can be directed to the corresponding author.
