# Supplementary material for: Impact of pediatric tracheostomy on family caregivers’ burden and quality of life: a systematic review and meta-analysis
Source: Front Public Health. 2025 Jan 15;12:1485544. doi: 10.3389/fpubh.2024.1485544 (PMC11780180; doi:10.3389/fpubh.2024.1485544)
Supplement: Supplementary file 1 [file Supplementary_file_1.docx]

**Supplementary Material 1: MOOSE Checklist**

| **Item No** | **Recommendation** | **Reported on Page No** |
| --- | --- | --- |
| Reporting of background should include | | |
| 1 | Problem definition | 5-6 |
| 2 | Hypothesis statement | NA |
| 3 | Description of study outcome(s) | 5-6 |
| 4 | Type of exposure or intervention used | 5-6 |
| 5 | Type of study designs used | 6 |
| 6 | Study population | 6-7 |
| Reporting of search strategy should include | | |
| 7 | Qualifications of searchers (e.g., librarians and investigators) | 6 |
| 8 | Search strategy, including time period included in the synthesis and key words | 6; supp material 2 |
| 9 | Effort to include all available studies, including contact with authors | 7 |
| 10 | Databases and registries searched | 6 |
| 11 | Search software used, name and version, including special features used (e.g., explosion) | 6 |
| 12 | Use of hand searching (e.g., reference lists of obtained articles) | 6 |
| 13 | List of citations located and those excluded, including justification | 7, Fig 1 |
| 14 | Method of addressing articles published in languages other than English | 6 |
| 15 | Method of handling abstracts and unpublished studies | 6 |
| 16 | Description of any contact with authors | 6 |
| Reporting of methods should include | | |
| 17 | Description of relevance or appropriateness of studies assembled for assessing the hypothesis to be tested | 6 |
| 18 | Rationale for the selection and coding of data (e.g., sound clinical principles or convenience) | 6 |
| 19 | Documentation of how data were classified and coded (e.g., multiple raters, blinding and interrater reliability) | 6-7 |
| 20 | Assessment of confounding (e.g., comparability of cases and controls in studies where appropriate) | - |
| 21 | Assessment of study quality, including blinding of quality assessors, stratification or regression on possible predictors of study results | 7 |
| 22 | Assessment of heterogeneity | 7 |
| 23 | Description of statistical methods (e.g., complete description of fixed or random effects models, justification of whether the chosen models account for predictors of study results, dose-response models, or cumulative meta-analysis) in sufficient detail to be replicated | 7 |
| 24 | Provision of appropriate tables and graphics | Tables 1-2, Figs 1-2, Supplementary Materials 1-5 |
| Reporting of results should include | | |
| 25 | Graphic summarizing individual study estimates and overall estimate | Fig 2, Supplementary Material 5 |
| 26 | Table giving descriptive information for each study included | Table 1 |
| 27 | Results of sensitivity testing (e.g., subgroup analysis) | NA |
| 28 | Indication of statistical uncertainty of findings | 6-7 |
