# Supplementary material for: Impact of pediatric tracheostomy on family caregivers’ burden and quality of life: a systematic review and meta-analysis
Source: Front Public Health. 2025 Jan 15;12:1485544. doi: 10.3389/fpubh.2024.1485544 (PMC11780180; doi:10.3389/fpubh.2024.1485544)
Supplement: Supplementary file 2 [file Supplementary_file_2.docx]

**Supplementary Material 2: Search Strategy**

***(i) PubMed***

| **Search number** | **Search string** |
| --- | --- |
| #1 | "pediatrics"[MeSH Terms] OR "pediatric*"[Title/Abstract] OR "paediatric*"[Title/Abstract] OR "infant"[MeSH Terms] OR "infant*"[Title/Abstract] OR "infanc*"[Title/Abstract] OR "newborn*"[Title/Abstract] OR "neonat*"[Title/Abstract] OR "baby"[Title/Abstract] OR "babies"[Title/Abstract] OR "child"[MeSH Terms] OR "child*"[Title/Abstract] OR "toddler*"[Title/Abstract] OR "kid"[Title/Abstract] OR "kids"[Title/Abstract] OR "adolescent"[MeSH Terms] OR "adolescen*"[Title/Abstract] OR "youth*"[Title/Abstract] OR "teen*"[Title/Abstract] OR "young person*"[Title/Abstract] OR "young adult"[MeSH Terms] OR "young adult"[Title/Abstract] |
| #2 | "tracheostomy"[MeSH Terms] OR "tracheostom*"[Title/Abstract] OR "tracheotomy"[MeSH Terms] OR "tracheotom*"[Title/Abstract] |
| #3 | “Quality of life” [MeSH Terms] or "Quality of life"[Title/Abstract] OR "Psychosocial"[Title/Abstract] OR "Karnofsky Performance Status"[Title/Abstract] OR "Life Quality"[Title/Abstract] OR "HRQOL"[Title/Abstract] OR "HRQL"[Title/Abstract] OR "PedsQL"[Title/Abstract] OR "QOL"[Title/Abstract] OR "caregiv*"[Title/Abstract] OR "care giv*"[Title/Abstract] OR "carer*"[Title/Abstract] OR "parent*"[Title/Abstract] OR "grandparent*"[Title/Abstract] OR "mother*"[Title/Abstract] OR "father*"[Title/Abstract] OR "Family"[Title/Abstract] OR "Families"[Title/Abstract] OR "guardian*"[Title/Abstract] OR "sibling*"[Title/Abstract] OR "Caregiver burden"[Title/Abstract] OR "Care burden"[Title/Abstract] OR "Psychological general well being index"[Title/Abstract] OR "PGWBI"[Title/Abstract] OR "Burden Scale for Family Caregivers"[Title/Abstract] OR "BSFC"[Title/Abstract] OR "Zarit Burden Interview"[Title/Abstract] OR "ZBI"[Title/Abstract] OR "Impact on family scale"[Title/Abstract] OR "IOFS"[Title/Abstract] OR "Caregiver Reaction Assessment"[Title/Abstract] OR "CRA"[Title/Abstract] OR "EuroQol"[Title/Abstract] OR "Burden Assessment Scale"[Title/Abstract] OR "BAS"[Title/Abstract] OR "Family burden scale"[Title/Abstract] OR "FBS"[Title/Abstract] OR "Family Burden Module"[Title/Abstract] OR "Family Burden Interview Schedule"[Title/Abstract] OR "FBIS"[Title/Abstract] OR "GHQ-28"[Title/Abstract] OR "Caregiver strain index"[Title/Abstract] OR "CSI"[Title/Abstract] OR "BASC"[Title/Abstract] OR "Caregiver Quality of Life Index"[Title/Abstract] OR "Experience of caregiving inventory"[Title/Abstract] OR "Neo five factor inventory"[Title/Abstract] OR "Perceived burden scale"[Title/Abstract] OR "PBS"[Title/Abstract] OR "Perceived caregiver burden"[Title/Abstract] OR "PCB"[Title/Abstract] OR "Relative stress scale"[Title/Abstract] OR "RSS"[Title/Abstract] OR "Subjective burden scale"[Title/Abstract] OR "SCB"[Title/Abstract] |
| #4 | #1 and #2 and #3 |

***(ii) Embase***

| **Search number** | **Search string** |
| --- | --- |
| #1 | 'pediatrics'/exp OR pediatric*:ab,ti OR paediatric*:ab,ti OR 'infant'/exp OR infant*:ab,ti OR infanc*:ab,ti OR newborn*:ab,ti OR neonat*:ab,ti OR baby:ab,ti OR babies:ab,ti OR 'child'/exp OR child*:ab,ti OR toddler*:ab,ti OR kid:ab,ti OR kids:ab,ti OR 'adolescent'/exp OR adolescen*:ab,ti OR youth*:ab,ti OR teen*:ab,ti OR 'young person*':ab,ti OR 'young adult'/exp OR 'young adult*':ab,ti |
| #2 | 'tracheostomy'/exp OR tracheostom*:ab,ti OR 'tracheotomy'/exp OR tracheotom*:ab,ti |
| #3 | 'quality of life'/exp or 'quality of life':ab,ti OR psychosocial:ab,ti OR 'karnofsky performance status':ab,ti OR 'life quality':ab,ti OR hrqol:ab,ti OR hrql:ab,ti OR pedsql:ab,ti OR qol:ab,ti OR caregiv*:ab,ti OR 'care giv*':ab,ti OR carer*:ab,ti OR parent*:ab,ti OR grandparent*:ab,ti OR mother*:ab,ti OR father*:ab,ti OR family:ab,ti OR families:ab,ti OR guardian*:ab,ti OR sibling*:ab,ti OR 'caregiver burden':ab,ti OR 'care burden':ab,ti OR 'psychological general well being index':ab,ti OR pgwbi:ab,ti OR 'burden scale for family caregivers':ab,ti OR bsfc:ab,ti OR 'zarit burden interview':ab,ti OR zbi:ab,ti OR 'impact on family scale':ab,ti OR iofs:ab,ti OR 'caregiver reaction assessment':ab,ti OR cra:ab,ti OR 'ama caregiver self-assessment questionnaire':ab,ti OR 'euroqol':ab,ti OR 'burden assessment scale':ab,ti OR bas:ab,ti OR 'family burden scale':ab,ti OR fbs:ab,ti OR 'family burden module':ab,ti OR 'family burden interview schedule':ab,ti OR 'family burden inventory scale':ab,ti OR fbis:ab,ti OR 'general healthcare questionnaire':ab,ti OR 'ghq-28':ab,ti OR 'caregiver strain index':ab,ti OR csi:ab,ti OR 'brief assessment scale for caregivers':ab,ti OR basc:ab,ti OR 'caregiver assessment scale':ab,ti OR 'caregiver quality of life index':ab,ti OR 'experience of caregiving inventory':ab,ti OR 'neo five factor inventory':ab,ti OR 'perceived burden scale':ab,ti OR pbs:ab,ti OR 'perceived caregiver burden':ab,ti OR pcb:ab,ti OR 'rapid screen for caregiver burden':ab,ti OR 'relative stress scale':ab,ti OR rss:ab,ti OR 'subjective and objective family burden interview':ab,ti OR 'subjective burden scale':ab,ti OR scb:ab,ti |
| #4 | #1 and #2 and #3 |

***(iii) Cochrane***

| **Search number** | **Search string** |
| --- | --- |
| #1 | ("pediatric*" OR "paediatric*" OR "infant*" OR "infanc*" OR "newborn*" OR "neonat*" OR "baby" OR "babies" OR "child*" OR "toddler*" OR "kid" OR "kids" OR "adolescen*" OR "youth*" OR "teen*" OR "young person*" OR "young adult"):ti,ab |
| #2 | MeSH descriptor: [Pediatrics] explode all trees |
| #3 | (“Quality of life” OR Psychosocial OR “Karnofsky Performance Status” OR “Life Quality” OR HRQOL OR HRQL OR PedsQL OR QOL OR Caregiv* OR “Care giv*” OR Carer* OR Parent* OR Grandparent* OR Mother* OR Father* OR Family OR Families OR Guardian* OR Sibling* OR "Caregiver burden” OR “Care burden” OR “Psychological general well being index” OR PGWBI OR “Burden Scale for Family Caregivers” OR BSFC OR “Zarit Burden Interview” OR ZBI OR “Impact on family scale” OR IOFS OR "Caregiver Reaction Assessment” OR CRA OR “AMA caregiver self-assessment questionnaire” OR “EuroQol” OR “Burden Assessment Scale” OR BAS OR “Family burden scale” OR FBS OR “Family Burden Module” OR “Family Burden Interview Schedule” OR “Family burden Inventory scale” OR FBIS OR “General Healthcare Questionnaire” OR “GHQ-28” OR  “Caregiver strain index” OR CSI OR “Brief assessment scale for caregivers” OR BASC OR “Caregiver assessment scale” OR “Caregiver Quality of Life Index” OR “Experience of caregiving inventory” OR “Neo five factor inventory” OR “Perceived burden scale” OR PBS OR “Perceived caregiver burden” OR PCB OR “Rapid screen for caregiver burden” OR “Relative stress scale” OR RSS OR “Subjective and objective family burden interview” OR “Subjective burden scale” OR SCB):ti,ab |
| #4 | MeSH descriptor: [Quality of Life] explode all trees |
| #5 | ("tracheostom*" OR "tracheotom*"):ti,ab |
| #6 | MeSH descriptor: [Tracheostomy] explode all trees |
| #7 | (#1 or #2) and (#3 or #4) and (#5 or #6) |

***(iv) CINAHL***

| **Search number** | **Search string** |
| --- | --- |
| #1 | TI ( Pediatric* OR Paediatric* OR   Infant* OR Infanc* OR Newborn* OR Neonat* OR Baby OR Babies OR   Child* OR Toddler* OR Kid OR Kids OR   Adolescen* OR Youth* OR Teen* OR “Young person*” OR   “Young adult*” ) OR AB ( Pediatric* OR Paediatric* OR   Infant* OR Infanc* OR Newborn* OR Neonat* OR Baby OR Babies OR   Child* OR Toddler* OR Kid OR Kids OR   Adolescen* OR Youth* OR Teen* OR “Young person*” OR   “Young adult*” ) OR MH ( Pediatrics OR Child OR Adolescence OR Young adult ) |
| #2 | TI ( Tracheostom* OR Tracheotom* ) OR AB ( Tracheostom* OR Tracheotom* ) OR MH tracheostomy |
| #3 | TI (“Quality of life” OR Psychosocial OR “Karnofsky Performance Status” OR “Life Quality” OR HRQOL OR HRQL OR PedsQL OR QOL OR Caregiv* OR “Care giv*” OR Carer* OR Parent* OR Grandparent* OR Mother* OR Father* OR Family OR Families OR Guardian* OR Sibling* OR "Caregiver burden” OR “Care burden” OR “Psychological general well being index” OR PGWBI OR “Burden Scale for Family Caregivers” OR BSFC OR “Zarit Burden Interview” OR ZBI OR “Impact on family scale” OR IOFS OR "Caregiver Reaction Assessment” OR CRA OR “AMA caregiver self-assessment questionnaire” OR “EuroQol” OR “Burden Assessment Scale” OR BAS OR “Family burden scale” OR FBS OR “Family Burden Module” OR “Family Burden Interview Schedule” OR “Family burden Inventory scale” OR FBIS OR “General Healthcare Questionnaire” OR “GHQ-28” OR  “Caregiver strain index” OR CSI OR “Brief assessment scale for caregivers” OR BASC OR “Caregiver assessment scale” OR “Caregiver Quality of Life Index” OR “Experience of caregiving inventory” OR “Neo five factor inventory” OR “Perceived burden scale” OR PBS OR “Perceived caregiver burden” OR PCB OR “Rapid screen for caregiver burden” OR “Relative stress scale” OR RSS OR “Subjective and objective family burden interview” OR “Subjective burden scale” OR SCB) OR AB ((“Quality of life” OR Psychosocial OR “Karnofsky Performance Status” OR “Life Quality” OR HRQOL OR HRQL OR PedsQL OR QOL OR Caregiv* OR “Care giv*” OR Carer* OR Parent* OR Grandparent* OR Mother* OR Father* OR Family OR Families OR Guardian* OR Sibling* OR "Caregiver burden” OR “Care burden” OR “Psychological general well being index” OR PGWBI OR “Burden Scale for Family Caregivers” OR BSFC OR “Zarit Burden Interview” OR ZBI OR “Impact on family scale” OR IOFS OR "Caregiver Reaction Assessment” OR CRA OR “AMA caregiver self-assessment questionnaire” OR “EuroQol” OR “Burden Assessment Scale” OR BAS OR “Family burden scale” OR FBS OR “Family Burden Module” OR “Family Burden Interview Schedule” OR “Family burden Inventory scale” OR FBIS OR “General Healthcare Questionnaire” OR “GHQ-28” OR  “Caregiver strain index” OR CSI OR “Brief assessment scale for caregivers” OR BASC OR “Caregiver assessment scale” OR “Caregiver Quality of Life Index” OR “Experience of caregiving inventory” OR “Neo five factor inventory” OR “Perceived burden scale” OR PBS OR “Perceived caregiver burden” OR PCB OR “Rapid screen for caregiver burden” OR “Relative stress scale” OR RSS OR “Subjective and objective family burden interview” OR “Subjective burden scale” OR SCB) OR MH ( "Quality of life") |
| #4 | #1 and #2 and #3 |

***(v) PsycINFO***

| **Search number** | **Search string** |
| --- | --- |
| #1 | (pediatric* or paediatric* or infant* or infanc* or newborn* or neonat* or baby or babies or child* or toddler* or kid or kids or adolescen* or youth* or teen* or "young person*" or "young adult*").ti,ab or exp pediatrics/ or exp early childhood development/ or exp early adolescence/ or exp emerging adulthood/ |
| #2 | (tracheostom* or tracheotom*).ti,ab. |
| #3 | (“Quality of life” OR Psychosocial OR “Karnofsky Performance Status” OR “Life Quality” OR HRQOL OR HRQL OR PedsQL OR QOL OR Caregiv* OR “Care giv*” OR Carer* OR Parent* OR Grandparent* OR Mother* OR Father* OR Family OR Families OR Guardian* OR Sibling* OR "Caregiver burden” OR “Care burden” OR “Psychological general well being index” OR PGWBI OR “Burden Scale for Family Caregivers” OR BSFC OR “Zarit Burden Interview” OR ZBI OR “Impact on family scale” OR IOFS OR "Caregiver Reaction Assessment” OR CRA OR “AMA caregiver self-assessment questionnaire” OR “EuroQol” OR “Burden Assessment Scale” OR BAS OR “Family burden scale” OR FBS OR “Family Burden Module” OR “Family Burden Interview Schedule” OR “Family burden Inventory scale” OR FBIS OR “General Healthcare Questionnaire” OR “GHQ-28” OR  “Caregiver strain index” OR CSI OR “Brief assessment scale for caregivers” OR BASC OR “Caregiver assessment scale” OR “Caregiver Quality of Life Index” OR “Experience of caregiving inventory” OR “Neo five factor inventory” OR “Perceived burden scale” OR PBS OR “Perceived caregiver burden” OR PCB OR “Rapid screen for caregiver burden” OR “Relative stress scale” OR RSS OR “Subjective and objective family burden interview” OR “Subjective burden scale” OR SCB).ti,ab. or exp quality of life/ |
| #4 | #1 and #2 and #3 |
