# Supplementary material for: Impact of pediatric tracheostomy on family caregivers’ burden and quality of life: a systematic review and meta-analysis
Source: Front Public Health. 2025 Jan 15;12:1485544. doi: 10.3389/fpubh.2024.1485544 (PMC11780180; doi:10.3389/fpubh.2024.1485544)
Supplement: Supplementary file 3 [file Supplementary_file_3.docx]

**Supplementary Resource 3: Newcastle- Ottawa scale for cross sectional studies**

| Author | Year | Selection Bias Assessment (Maximum 5 stars) | | | | Comparability (Maximum 2 stars) | Outcome (Maximum 3 stars) | | Total score (Maximum 10 stars) | Quality of study^[[1]](#footnote-1)^ |
| --- | --- | --- | --- | --- | --- | --- | --- | --- | --- | --- |
|  |  | Representativeness of the sample | Sample size | Non-respondents | Ascertainment of the exposure (risk factor) | Confounding factors are controlled | Assessment of the outcome | Statistical Test |  |  |
| Chandran et al | 2021 | 1 | 1 | 1 | 2 | 2 | 1 | 1 | 9 | Very good |
| Din et al | 2020 | 1 | 1 | 0 | 2 | 2 | 1 | 1 | 8 | Good |
| Johnson et al | 2021 | 1 | 1 | 0 | 2 | 2 | 1 | 1 | 8 | Good |
| Westwood et al | 2019 | 1 | 1 | 0 | 2 | 2 | 1 | 1 | 8 | Good |
| Mirza et al | 2022 | 1 | 1 | 0 | 2 | 2 | 1 | 1 | 8 | Good |
| Wynings et al | 2023 | 1 | 1 | 0 | 2 | 2 | 1 | 1 | 8 | Good |
| Hartnick et al | 2003 | 1 | 1 | 0 | 1 | 2 | 1 | 1 | 7 | Good |
| Hopkins et al | 2009 | 1 | 1 | 1 | 2 | 0 | 1 | 1 | 7 | Good |
| Fuyuki et al | 2021 | 1 | 1 | 1 | 2 | 2 | 1 | 1 | 9 | Very good |
| Mavi et al | 2021 | 1 | 1 | 1 | 2 | 2 | 1 | 1 | 9 | Very good |
| Montagnino et al | 2004 | 1 | 1 | 0 | 2 | 2 | 1 | 1 | 8 | Good |
| Salley et al | 2021 | 1 | 1 | 0 | 2 | 0 | 1 | 1 | 6 | Satisfactory |
| Settoon et al | 2021 | 1 | 1 | 0 | 2 | 2 | 1 | 1 | 8 | Good |
| Yotani et al | 2014 | 1 | 1 | 1 | 2 | 2 | 1 | 1 | 9 | Very good |
| Gursoy et al | 2022 | 1 | 1 | 0 | 2 | 2 | 1 | 1 | 8 | Good |
| Joseph et al | 2014 | 1 | 1 | 0 | 1 | 2 | 1 | 1 | 7 | Good |
| Singer et al | 1989 | 1 | 1 | 0 | 2 | 2 | 1 | 1 | 8 | Good |
| Baddour et al | 2021 | 1 | 1 | 0 | 2 | 2 | 1 | 1 | 8 | Good |
| Liao et al | 2021 | 1 | 1 | 0 | 2 | 2 | 1 | 1 | 8 | Good |
| October et al | 2020 | 1 | 1 | 1 | 2 | 2 | 1 | 1 | 9 | Very good |
| Al-Faleh et al | 2023 | 1 | 1 | 0 | 2 | 2 | 1 | 1 | 8 | Good |
| Koker et al | 2023 | 1 | 1 | 1 | 2 | 2 | 1 | 1 | 9 | Very good |
| Verstraete et al | 2023 | 1 | 1 | 1 | 2 | 2 | 1 | 1 | 9 | Very good |

1. Unsatisfactory (0-4 points), Satisfactory (5-6 points), Good (7-8 points), Very good (9-10 points) [↑](#footnote-ref-1)
