# Supplementary material for: Impact of pediatric tracheostomy on family caregivers’ burden and quality of life: a systematic review and meta-analysis
Source: Front Public Health. 2025 Jan 15;12:1485544. doi: 10.3389/fpubh.2024.1485544 (PMC11780180; doi:10.3389/fpubh.2024.1485544)
Supplement: Supplementary file 4 [file Supplementary_file_4.docx]

Supplementary Resource 4: Pooled Analyses for PedsQL Family Impact Module (FIM) Subdomains

| ***(i) Physical Functioning Score***  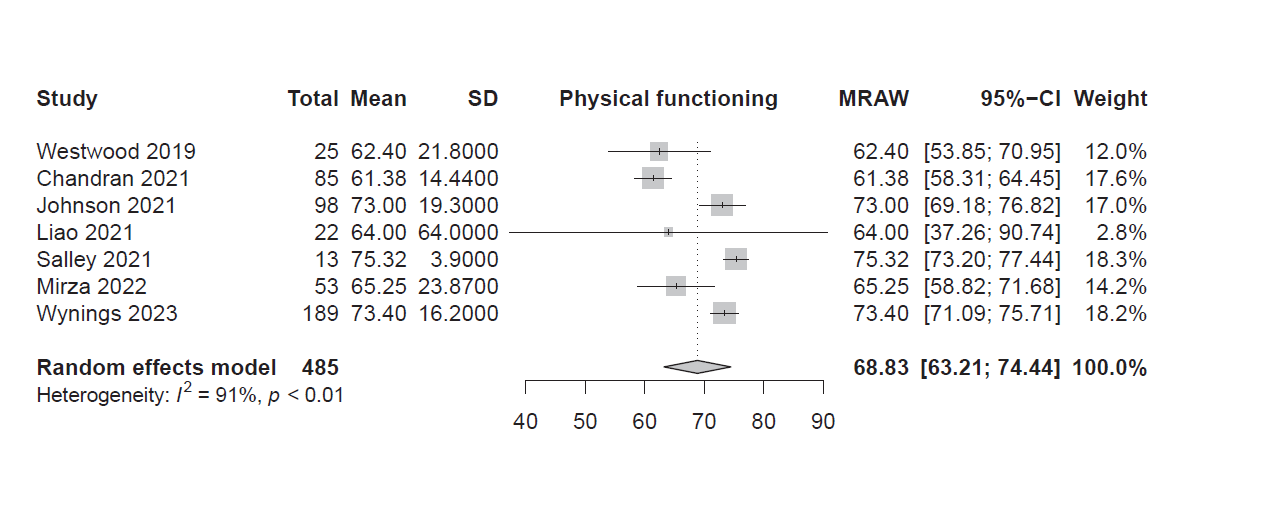 |
| --- |
| ***(ii) Emotional Functioning Score***  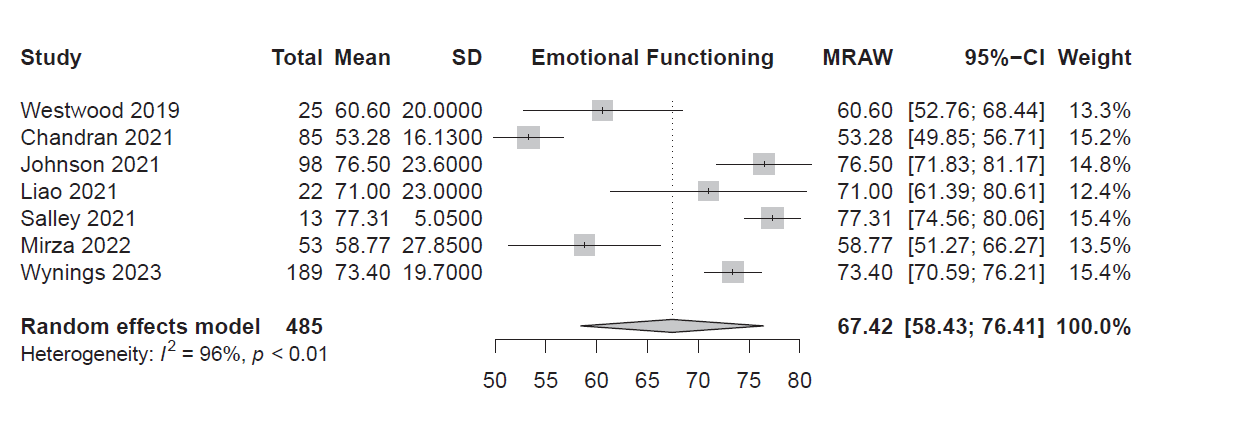 |
| ***(iii) Social Functioning Score***  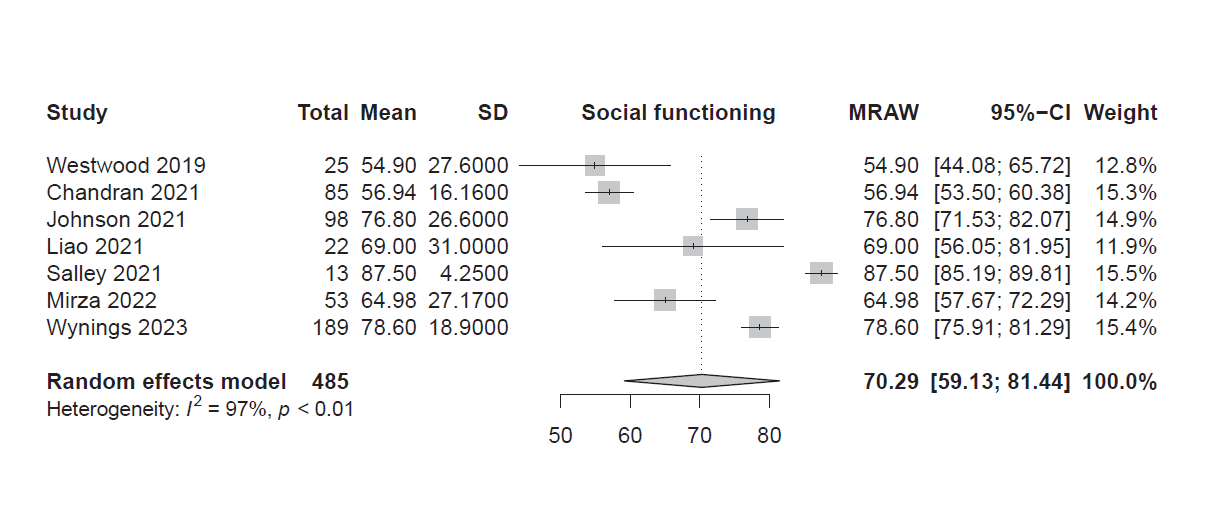 |
| ***(iv) Cognitive Functioning Score***  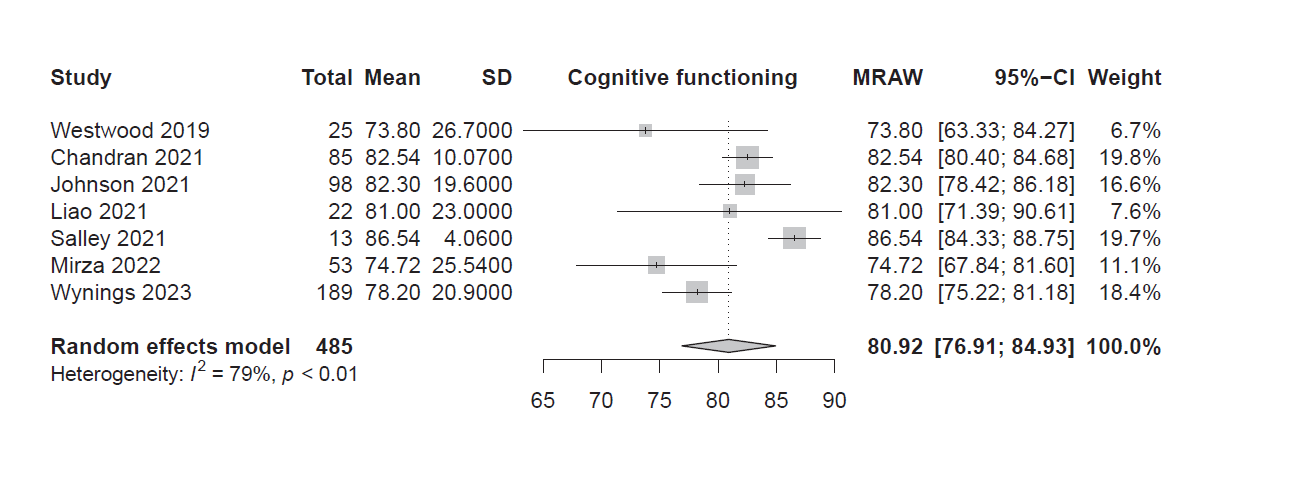 |
| ***(v) Communication Score***  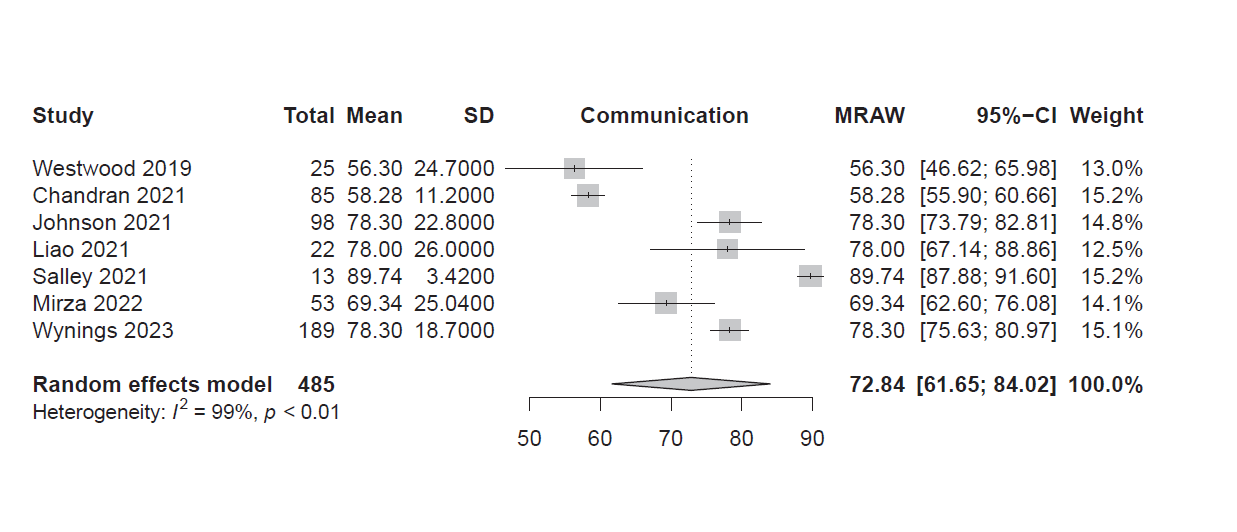 |
| ***(vi) Worry Score***  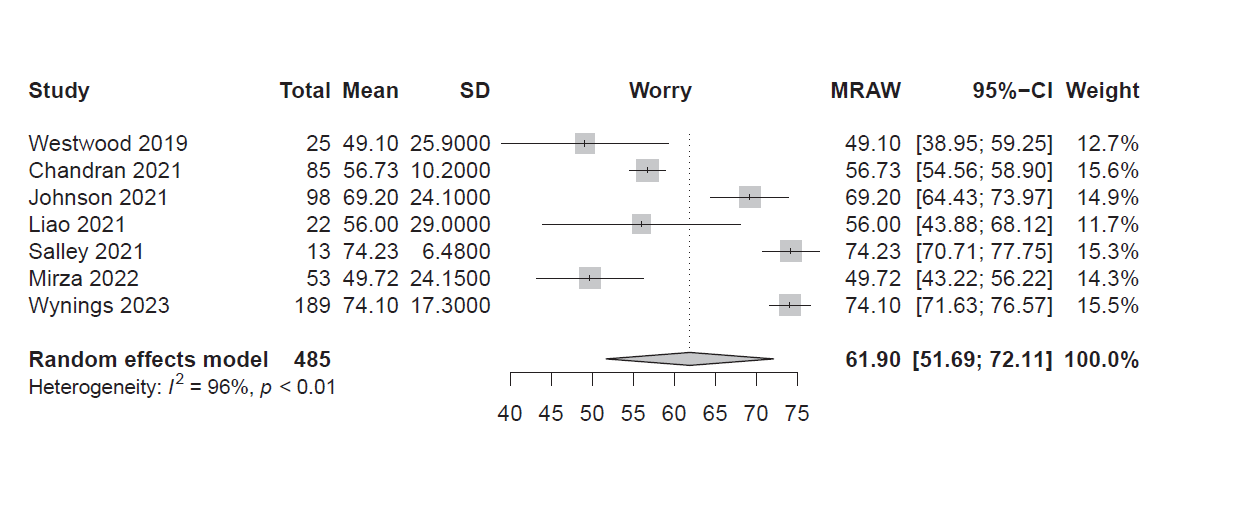 |

| ***(vii) Daily Activities Score***  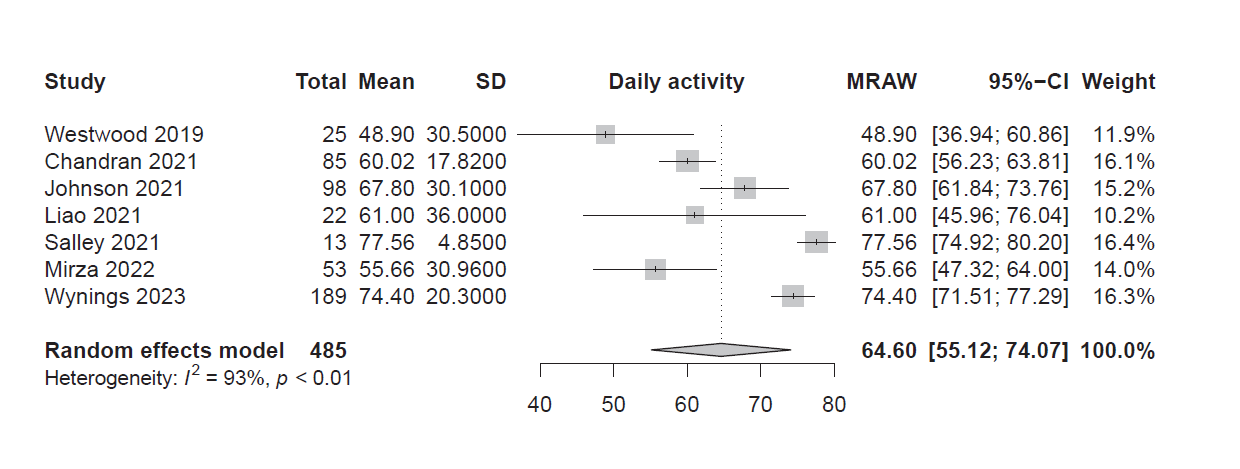 |
| --- |
| ***(viii) Family Relationships Score***  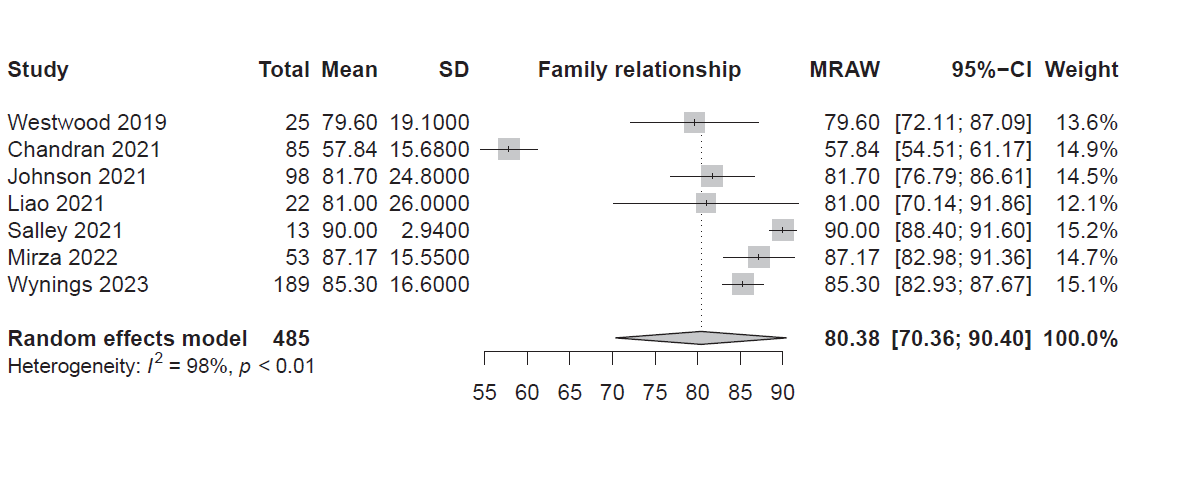 |
