# Supplementary material for: Impact of pediatric tracheostomy on family caregivers’ burden and quality of life: a systematic review and meta-analysis
Source: Front Public Health. 2025 Jan 15;12:1485544. doi: 10.3389/fpubh.2024.1485544 (PMC11780180; doi:10.3389/fpubh.2024.1485544)
Supplement: Supplementary file 5 [file Supplementary_file_5.docx]

Supplementary Material 5: PedsQL Family Impact Module (FIM) Scores of Caregivers of children with chronic conditions

|  | Tracheostomy (meta-analysis) | Sickle cell disease  (SCD) (1) | | Child with renal transplant (2) | Chronic pain (3) | | Children with cancer undergoing chemotherapy (4) | | Nephrotic syndrome  (5) | Asthma (6) | Heart disease (6) |
| --- | --- | --- | --- | --- | --- | --- | --- | --- | --- | --- | --- |
| Age of children  Median, Mean, number in age range |  | Median 10.0 (5.0, 14.0) | | Median 14.6 (4.4–17.8) | Mean 13.74 (2.71) | | 2-4 yr: 33  5-7yr: 16  8-12yr: 28  13-18yr: 18 | | Mean 6.6 (3.17) | 2-4yr: 59  5-7yr: 42  8-12yr: 31  13-18yr: 4 | 2-4yr: 116  5-7yr: 61  8-12yr: 43  13-18yr: 44 |
| Total Family Impact Score | Mean (95% CI): 70.29 (61.20-79.37) | Mild SCD  Mean 79.1(18.3) | Severe SCD  Mean 71.53 (20.0) | Mean 74.8 (19.9) | Mothers  Mean 64.68 (19.54) | Fathers  Mean  67.89 (17.88) | In-patient:  Mean  67.60 (13.53) | Out-patient:  Mean  56.43 (16.27) | Mean 72.4 (22.6) | Mean 78.01 (18.2) | Mean 71.9 (22.1) |
| Parent HRQOL Score  Mean (95% CI or SD) | 69.27 (95% CI 60.88-77.67) | Mean 79.1 (21.2) | Mean 72.7 (18.9) | Mean 72.1 (19.5) | Mean 67.44 (20.73) | Mean 73.47 (17.98) | Mean 72.20 (13.86) | Mean 62.18 (17.07) | Mean 72.7 (24.1) | Mean 78.23 (21.3) | Mean 73.4 (23.1) |
| Family Functioning Score  Mean (95% CI or SD) | 72.96 (95% CI 65.92-80.00) | Mean 81.2 (22.7) | Mean 75 (28.6) | Mean 77.2 (23.6) | Mean 66.10 (23.41) | Mean 65.14 (23.31) | Mean 67.46 (21.77) | Mean 56.25 (22.35) | Mean 75.0 (21.4) | Mean 82.8 (16.4) | Mean 76.0 (25.6) |
| Physical Functioning  Mean (95% CI or SD) |  | Mean 69.4667 (32.0) | Mean 65.3 (20.7) |  |  |  |  |  | Mean 71.5 (23.7) | Mean 76.04 (27.3) | Mean 75 (24.9) |
| Emotional Functioning  Mean (95% CI or SD) |  | Mean 83.3 (23.0) | Mean 73.3 (22.9) |  |  |  |  |  | Mean 73.3 (38.0) | Mean 78.3 (26.3) | Mean 73.3 (26.1) |
| Social Functioning  Mean (95%CI or SD) |  | Mean 87.5 (24.0) | Mean 82.3 (31.0) |  |  |  |  |  | Mean 79.1 (33.3) | Mean 81.25 (28.1) | Mean 75 (28.0) |
| Cognitive Functioning  Mean (95% CI or SD) |  | Mean 84.1667 (30.6) | Mean 73.6 (27.3) |  |  |  |  |  | Mean 75 (26.6) | Mean 80 (30.0) | Mean 76.7 (26.1) |
| Communication  Mean (95% CI or SD) |  | Mean 87.5 (25.5) | Mean 75 (31.8) |  |  |  |  |  | Mean 83.3 (25.3) | Mean 91.7 (18.7) | Mean 77.8 (31.1) |
| Worry  Mean (95% CI or SD) |  | Mean 75.8 (38.3) | Mean 58.3 (30.5) |  |  |  |  |  | Mean 61.7 (30.4) | Mean 66.7 (28.1) | Mean 61.25 (25.2) |
| Daily Activities  Mean (95% CI or SD) |  | Mean 77.7 (31.9) | Mean 69.5 (31.8) |  |  |  |  |  | Mean 65.3 (34.8) | Mean 66.7 (25.0) | Mean 69.4 (31.1) |
| Family Relationships  Mean (95% CI or SD) |  | Mean 82.5 (28.7) | Mean 80 (30.5) |  |  |  |  |  | Mean 82.5 (28.5) | Mean 90 (18.7) | Mean 81.7 (26.1) |

NB: The median values from original articles were converted to mean and SD according to methods prescribed by Wang W, Liu J, et al 2104.(7)

References

1. Panepinto JA, Hoffmann RG, Pajewski NM. A psychometric evaluation of the PedsQL^TM^ Family Impact Module in parents of children with sickle cell disease. Health Qual Life Outcomes. 2009 Dec;7(1):32.

2. Lau KK, Giglia L, Chan H, Chan AK. Management of children after renal transplantation: highlights for general pediatricians. Transl Pediatr. 2012 Jul;1(1):35–46.

3. Jastrowski Mano KE, Khan KA, Ladwig RJ, Weisman SJ. The impact of pediatric chronic pain on parents’ health-related quality of life and family functioning: reliability and validity of the PedsQL 4.0 Family Impact Module. J Pediatr Psychol. 2011 Jun;36(5):517–27.

4. Scarpelli AC, Paiva SM, Pordeus IA, Ramos-Jorge ML, Varni JW, Allison PJ. Measurement properties of the Brazilian version of the Pediatric Quality of Life Inventory (PedsQL) cancer module scale. Health Qual Life Outcomes. 2008 Jan 22;6:7.

5. Mishra K, Ramachandran S, Firdaus S, Rath B. The impact of pediatric nephrotic syndrome on parents′ health-related quality of life and family functioning: An assessment made by the PedsQL 4.0 family impact module. Saudi J Kidney Dis Transplant. 2015;26(2):285.

6. Chen R, Hao Y, Feng L, Zhang Y, Huang Z. The Chinese version of the Pediatric Quality of Life Inventory^TM^ (PedsQL^TM^) Family Impact Module: cross-cultural adaptation and psychometric evaluation. Health Qual Life Outcomes. 2011 Mar 23;9:16.

7. Wan X, Wang W, Liu J, Tong T. Estimating the sample mean and standard deviation from the sample size, median, range and/or interquartile range. BMC Med Res Methodol. 2014 Dec;14(1):135.
